# Cloning and Characterization of Two Iridoid Synthase Homologs from *Swertia Mussotii*

**DOI:** 10.3390/molecules22081387

**Published:** 2017-08-22

**Authors:** Beibei Xiang, Xiaoxue Li, Yan Wang, Xiaoxuan Tian, Zhen Yang, Lin Ma, Xia Liu, Yong Wang

**Affiliations:** 1 School of Chinese Materia Medica, Tianjin University of Traditional Chinese Medicine, Anshan road 312, Tianjin 300193, China; paozhijiaoxue@126.com (Y.W.); yzwygb@126.com (Z.Y.); malin7983@163.com (L.M.); 2 College of Life Science, Nankai University, Weijin road 94, 300071 Tianjin, China; m15822139395@163.com; 3 Tianjin State Key Laboratory of Modern Chinese Medicine, Tianjin University of Traditional Chinese Medicine, Anshan road 312, Tianjin 300193, China; tian_xiaoxuan@tjutcm.edu.cn; 4 Key Laboratory of Food Nutrition and Safety, Tianjin University of Science and Technology, Ministry of Education, No. 29, 13th Street, TEDA 300457, Tianjin, China

**Keywords:** *Swertia mussotii*, medicinal plant, secoiridoid biosynthesis, iridoid synthase, progesterone 5-β-reductase, heterologous expression, functional characterization

## Abstract

*Swertia mussotii* is an important medicinal plant found on the Qinghai Tibetan Plateau that has great economic and medicinal value. This plant has enjoyed a long history of use as a curative for hepatitis. The biological activity of secoiridoids, including gentiopicroside and swertiamarin, has been mainly tested for its anti-hepatitis effects. Here, we identify two candidate genes (*SmIS1* and *SmIS2*) that are homologues of iridoid synthase and that are components of the secoiridoid pathway in *S. mussotii*. Using sequencing and phylogenetic analyses, we confirm that SmIS1 and SmIS2 contain six conserved short-chain dehydrogenases/reductase (SDR) motifs and thus belong to the P5βRs group. The two purified *Escherichia coli*-expressed proteins reduced 8-oxogeranial to both nepetalactol and iridodials. A comparison of the kinetic parameters of SmIS1 and SmIS2 recombinant proteins revealed that SmIS2 has a lower affinity than SmIS1 for 8-oxogeranial. Transcript levels of the two genes were analysed in three different tissues of *S. mussotii* using semi-quantitative RT-PCR and RT-qPCR. SmIS1 and SmIS2 expression levels were more abundant in leaves and stems. This investigation adds to our knowledge of *P5βRs* genes in the secoiridoid synthesis pathway and provides candidate genes for genetically improving *S. mussotii* by enhancing secondary metabolite production.

## 1. Introduction

*Swertia mussotii* Franch belongs to the Gentianaceae family and grows on the Qinghai Tibetan Plateau at an altitude of greater than 3800 m [1,2]. The entire *S. mussotii* plant is used in Tibetan medicine known as Zang-Yin-Chen in Chinese. Zang-Yin-Chen has been widely used to treat diseases, such as liver disease and blood disease [3]. To date, a diverse array of pharmaceutically active compounds have been isolated from the *S. mussotii* plant [4], including xanthones and their derivatives, flavonoids, terpenoids, iridoids and secoiridoid glycosides, such as swertiamarin and gentiopicroside. 

Gentiopicroside has been assessed to confirm its anti-inflammatory, analgesic, and anti-hepatitis effects [5,6], whereas swertiamarin has been reported to have anti-hepatitis [7], anti-cancer [8], anti-diabetics [9] and anti-arthritic [10] activities. These results undoubtedly demonstrate the importance of *S. mussotii* as a medicinal plant. Therefore, biological research concerning secondary metabolism in *S. mussotii* should be strengthened. The biosynthesis of swertiamarin and related compounds in *Swertia chirayita* has been investigated and some of the enzymes involved in the biosynthetic pathway have been reported [11].

**Figure 1:**
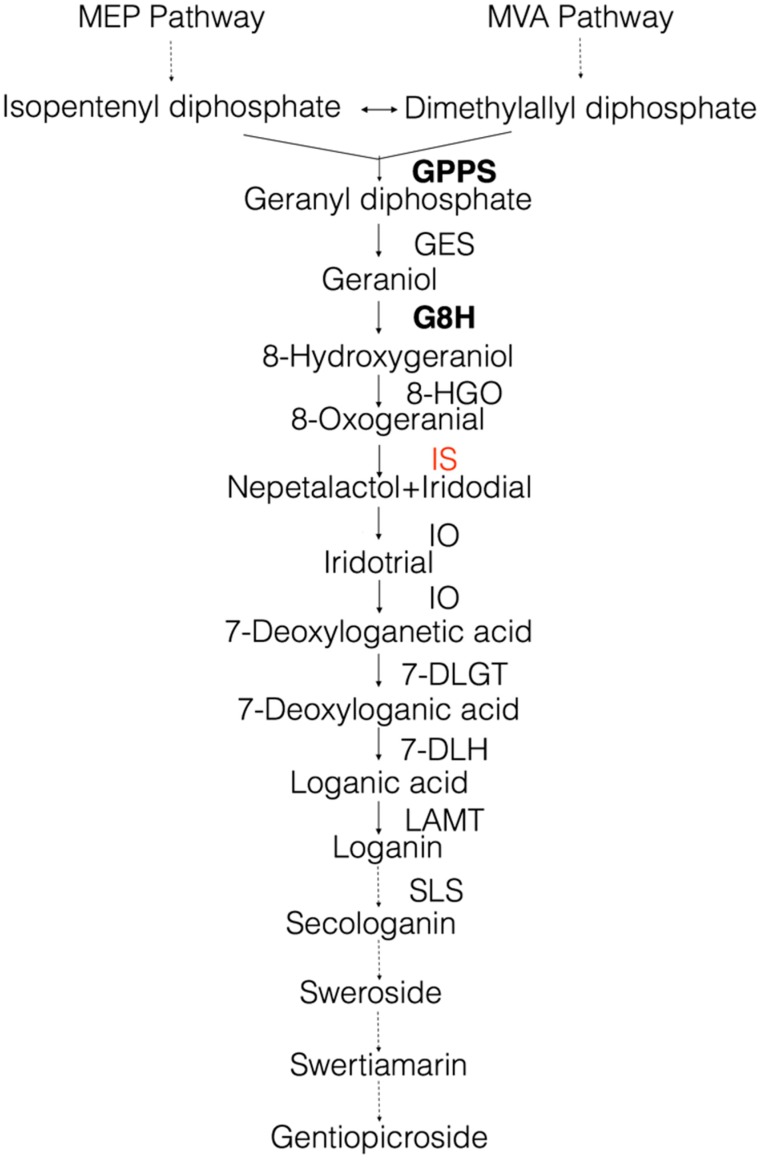
Schematic representation of the proposed in secoiridoid pathway. GPPS, geranyl diphosphate synthase; GES, geraniol synthase; G8H, geraniol 8-hydroxylase; 8-HGO, 8-hydroxygeraniol oxidoreductase; IS, iridoid synthase; IO, iridoid oxidase; 7-DLGT, 7-deoxyloganetic acid glucosyltransferase; 7-DLH, 7-deoxyloganic acid hydroxylase; LAMT, loganic acid O-methyltransferase; SLS, secologanin synthase. Genes in bold letters were previously cloned in *S. mussotii*; the gene in red was identified in this study.

At present, only one gene involved in swertiamarin biosynthesis has been characterized in *S. mussotii* [2]. To characterize other genes involved in this biosynthetic pathway in this important medicinal plant, we obtained the transcriptome in leaves of *S. mussotii* using RNA-sequencing. Based on the RNA-seq data and sequence similarity comparison with genes of *Catharanthus roseus*, we proposed assessing the secoiridoid biosynthesis pathway in *S. mussotii,* in which swertiamarin and gentiopicroside are processed via a classical MVA/MEP route of terpene biosynthesis followed by the secoiridoid pathway (Figure 1) [12]. Iridoid biosynthesis is initiated from geranyl diphosphate (GPP), a condensation product of isopentenyl diphosphate (IPP) and dimethylallyl diphosphate (DMAPP). Secoiridoids are derived from iridoids via the opening of a cyclopentane ring. In this study, we were particularly interested in characterization of the gene(s) encoding iridoid synthase (IS) because the enzyme catalyses the crucial step in the biosynthesis of iridoids [13]. IS belongs to the plant progesterone 5β-reductase (P5βR) family and uses the linear monoterpene 8-oxogeranial as substrate, probably couples an initial NAD(P)H-dependent reduction step with a subsequent cyclization step via a Michael reaction [14]. Recently, it was reported that iridoid synthase activity is common among the plant progesterone 5β-reductases and two (CrIS and CrP5βR4) of six *C. roseus* P5βR genes may participate in secoiridoid biosynthesis [15]. In our RNA-seq data, only three P5βR gene homologs were recognized. We cloned the full-length cDNA of two genes, SmIS1 and SmIS2. After hetero expression in *E. coli*, we compared the structure by homology modelling and catalytic properties. We also compared the expression of these two genes in different organs.

## 2. Results

### 2.1. Cloning, Sequencing and Phylogenetic Analysis of SmIS1 and SmIS2 cDNAs

The SmIS1 and SmIS2 gene sequences were retrieved from whole transcriptome data for *S. mussotii* using a BLAST similarity search program (http://www.ncbi.nlm.nih.gov/BLAST). The cDNA was cloned and its sequence was analysed using total RNA isolated from young leaves of the *S. mussotii* plant. The cDNA was used as a template for PCR, which was performed using primers designed for conserved regions in the 5′ and 3′ ends of the open reading frame (ORF). The sequences were submitted to GenBank and assigned accession numbers MF044036 (SmIS1) and MF044037 (SmIS2). The putative SmIS1 cDNA contained an ORF of 1173 bp that encoded a protein with 390 amino acid residues, and the putative SmIS2 cDNA contained an ORF of 1170 bp that encoded a protein with 389 amino acid residues. The calculated molecular mass and estimated pI of SmIS1 were 44.2 kDa and 6.08, respectively. The calculated molecular mass and estimated pI of SmIS2 were 43.9 kDa and 5.60, respectively.

To calculate the amino acid sequence identity of the two proteins and other orthologues, multiple alignments were performed. SmIS1 and SmIS2 sequences were highly similar to those of CrIS and OeIS. SmIS1 shared 76.47% and 70.05% identity with CrIS and OeIS, respectively. SmIS2 shared 58.26% and 58.29% identity with CrIS and OeIS, respectively.

**Figure 2:**
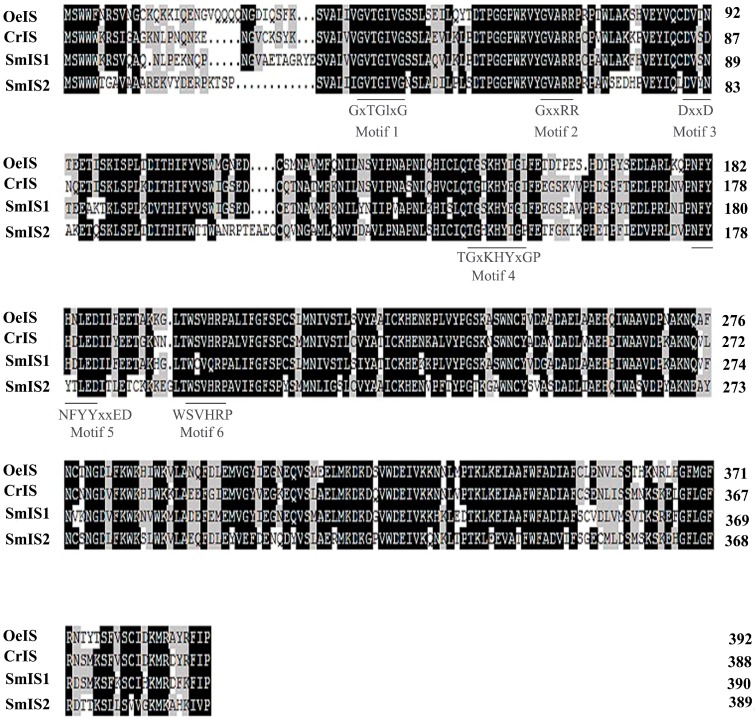
Amino acid sequence comparison of SmIS1 and Smis2 with other ISs. CrIS (*C. roseus*, GenBank: AFW98981.1); OeIS (*O. europaea*, GenBank: ALV83438.1). The conserved motifs of the P5βRs are indicated with bars. The highly conserved residues are highlighted in black, whereas similar residues are highlighted in grey.

**Figure 3:**
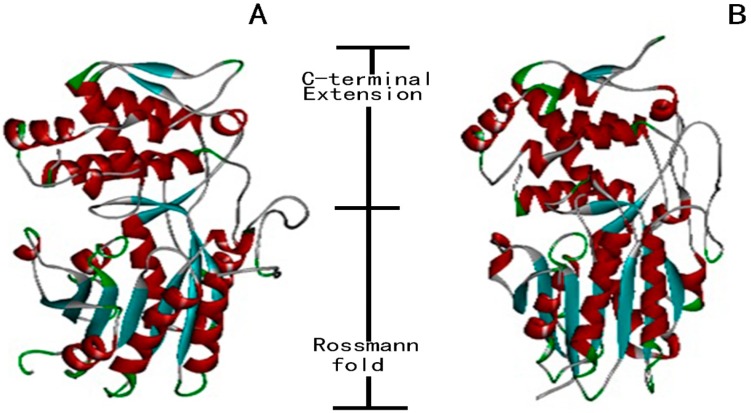
Predicted three-dimensional homology structure of SmIS1 (**A**) and SmIS2 (**B**) using recombinant CrIS from *C. roseus* (5COB) as a template. Helices and sheets are represented in red and blue, respectively.

The sequence analysis confirmed that SmIS1 and SmIS2 contained six conserved short-chain dehydrogenase/reductase (SDR) motifs and that these proteins therefore belong to the P5βRs group [16], which also includes CrIS and OeIS. Tyr180 (178) and Lys178 (146) were observed in the conserved motifs of SmIS1 and SmIS2, respectively. These residues may control the enzymatic activity and substrate specificity of the proteins (Figure 2). The 3D structures of the proteins were modelled based on plant P5βRs crystal structures that were available in the Protein Database (http://www.rcsb.org/pdb/home/home.do). Analyses of their entire lengths revealed that SmIS1 and SmIS2 were 57–78% identical to *D. lanata* 2V6G and *C. roseus* 5COB, which have solved protein structures. Using *C. roseus* CrIS (PDB: 5COB) as a template for homology modelling, we generated 3D models of SmIS1 and SmIS2 (Figure 3). The SmIS1 model was 36.15% a-helices, 10.26% β-strands, and 53.59% loop structures. The SmIS2 model was 35.99% a-helices, 11.31% β-strands, and 52.70% loop structures. Furthermore, the GMQE scores of the modelled SmIS1 and SmIS2 were 0.89 and 0.80, respectively, and their QMEAN Z-Scores were −1.29 and −1.88, respectively. These data confirm that the structural models are robust and potentially useful for generating hypotheses about their substrates.

**Figure 4:**
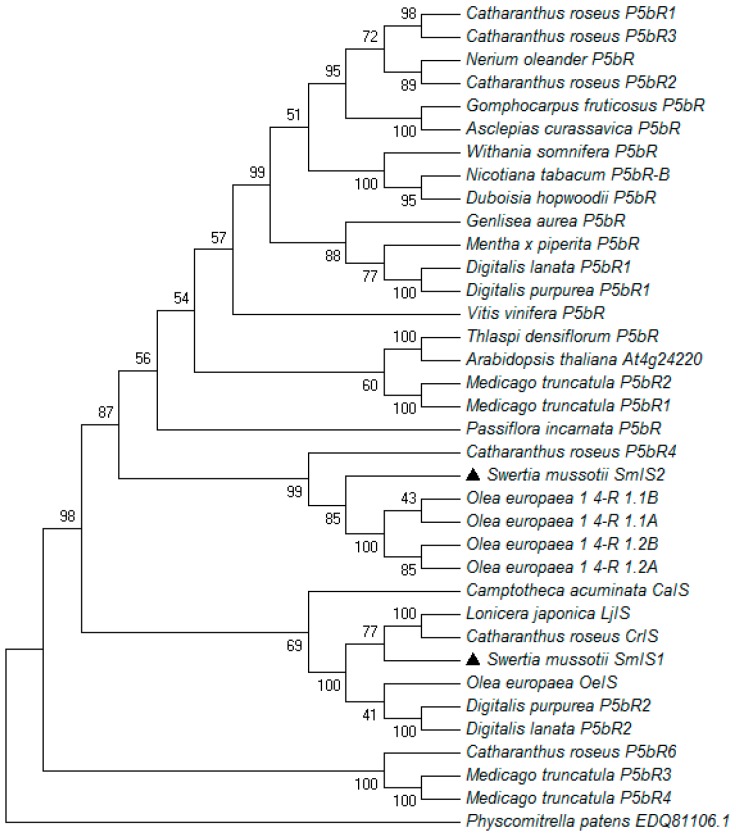
Phylogenetic tree of SmIS1 and SmIS2 together with 34 known P5βRs. The phylogenetic tree was drawn using *P. patens* (GenBank: EDQ81106.1) as an outgroup and the following amino acid sequences with accession IDs noted in brackets: *C. roseus* P5bR1 (AIW09143.1); *C. roseus* P5bR3(AIW09145.1); *Nerium oleander* P5bR-B (ADG56540.1); *C. roseus* P5bR2 (AIW09144.1); *Gomphocarpus fruticosus* P5bR (ADG56546.1); *Asclepias curassavica* P5bR (ADG56538.1); *Withania somnifera* P5bR (AEY82379.1); *Nicotiana tabacum* P5bR (BAH47641.1); *Duboisia hopwoodii* P5bR (AFZ41795.1); *Genlisea aurea* P5bR (EPS65468.1); *Mentha x piperita* P5bR (ADG46022.1); *Digitalis lanata* P5bR1(AAS93804.1); *Digitalis purpurea* P5bR1(AAS93805.1); *Vitis vinifera* P5bR (ALB78111.1); *Thlaspi densiflorum* P5bR (ALD83449.1); *Arabidopsis thaliana* At4g24220 (NP_001078438.1); *Medicago truncatula* P5bR1 (AIW09149.1); *Medicago truncatula* P5bR2 (AIW09150.1); *Passiflora incarnata* P5bR (AFW16644.1); *C. roseus* P5bR 4 (AIW09146.1); *Olea europaea* 1,4-R 1.1B (ALV83440.1); *Olea europaea* 1,4-R 1.1A (ALV83439.1); *Olea europaea* 1,4-R 1.2B (ALV83442.1); *Olea europaea* 1,4-R 1.2A (ALV83441.1); *Camptotheca acuminata* CaIS (AON76722.1); *Lonicera japonica* LjIS (AMB61018.1); *C. roseus* CrIS (AFW98981.1); *Olea europaea* OeIS (ALV83438.1); *D. purpurea* P5bR2 (ACZ66261.1); *D. lanata* P5bR2 (ADL28122.1); *C. roseus* P5bR6 (AIW09148.1); *Medicago truncatula* P5bR3 (AIW09151.1); *Medicago truncatula* P5bR3 (AIW09152.1).

We performed a phylogenetic analysis using the amino acid sequences of SmIS1 and SmIS2 and their homologues in other plants (Figure 4). All of the compared species belonged to the P5βRs group. SmIS2 belonged to one subclade that clustered with *C. roseus* P5bR4 (AIW09146.1), *O. europaea* 1,4-R1.1B (ALV83440.1), *O. europaea* 1,4-R1.2B (ALV83442.1), *O. europaea* 1,4-R1.2A (ALV83441.1), and *O. europaea* 1,4-R1.1A (ALV83439.1). However, SmIS1 belonged to another subgroup that clustered with *C. acuminata* CaIS (AON76722.1), *L. japonica* LjIS (AMB61018.1), *C. roseus* CrIS (AFW98981.1), *O. europaea* OeIS (ALV83438.1), *D. purpurea* P5bR2 (ACZ66261.1), and *D. lanata* P5bR2 (ADL28122.1). This result is similar to that reported by Alagna et al. [17], who suggested that iridoid synthases potentially originated from an ancestor exclusively common to Asterids.

### 2.2. Functional Characterization and Substrate Preferences of Recombinant SmIS1 and SmIS2

**Figure 5:**
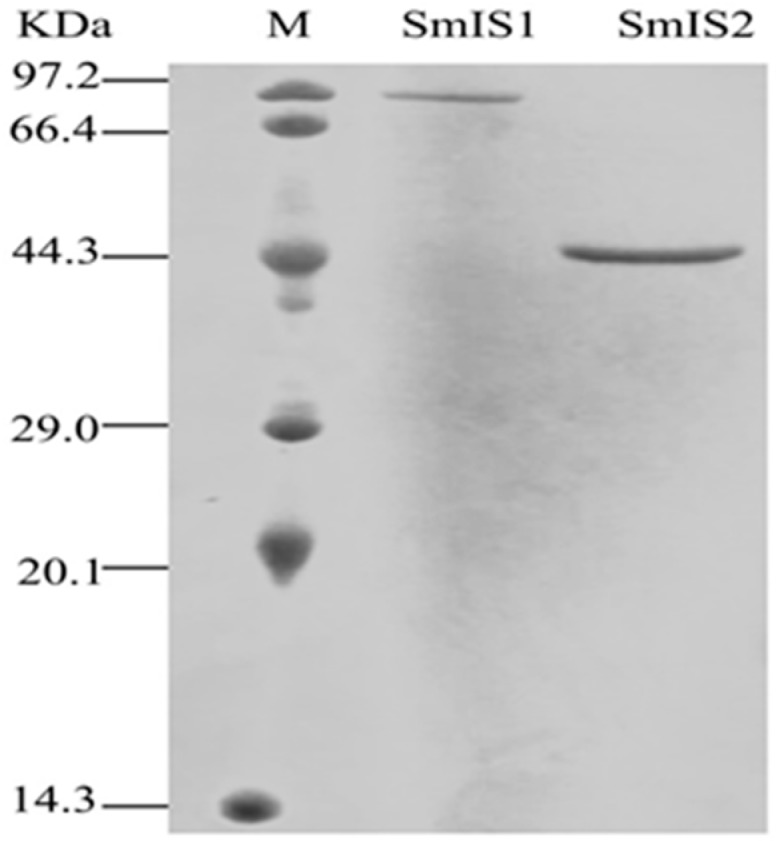
Heterologous expression of SmIS1 and Smis2 cDNA in *E. coli*. Lane SmIS1, purified recombinant SmIS1; lane SmIS2, purified recombinant SmIS2.

The full-length ORF of *SmIS*1 cDNA was subcloned into pETMALc-H. This vector was used to express a fusion protein with a maltose-binding protein at the N-terminus. The full-length ORF of SmIS2 cDNA was subcloned into pET-28a to produce a fusion protein with 6-His tags at both the *N*-terminus and *C*-terminus. Regarding SmIS1 heteroexpression, we also subcloned the SmIS1 ORF cDNA into the pET-28a vector to produce a fusion protein with His tags, which is a smaller tag. However, the SmIS1 recombinant protein always accumulated in inclusion bodies. Recombinant enzymes were produced in sufficient amounts to allow further characterization of SmIS1 and SmIS2. Affinity purification of both recombinant proteins resulted in single bands of the expected sizes that were visible on Coomassie-stained SDS-PAGE gels. A ~88 kDa protein band was observed in the SmIS1 lane (including 10 histidines and a MBP protein), and a ~46 kDa protein was observed in the SmIS2 lane (including two 6 histidines) (Figure 5). These findings indicated that SmIS1 and SmIS2 were successfully expressed in *E. coli* cells.

**Figure 6:**
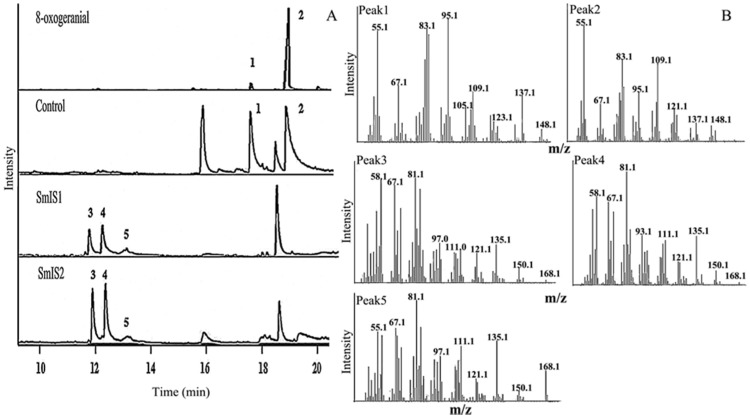
Functional characterization of SmIS1 and Smis2. (**A**) Analysis of the reaction product by GC-MS. Control, the reaction in the presence of denatured SmIS1 or SmIS2 proteins; (**B**) MS spectra of the substrates (peaks 1 and 2) and products (peaks 3, 4, and 5).

To determine whether recombinant SmIS1 and SmIS2 were both catalytically active, as we predicted, an in vitro assay to determine their enzyme activities and to detect their products was performed. We used 8-oxogeranial as a substrate for the enzyme assays in GC-MS. The enzymes were incubated with substrates in the presence of NADPH. Based on their mass, elemental composition, and fragmentation pattern and the enzyme activity of other known IS [13,15], the products were characterized as nepetalactol and iridodials (Figure 6: peak 3, peak 4 and peak 5). The substrate was a mixture of 8-oxoneral (peak 1) and 8-oxogeranial (peak 2). SmIS1 and SmIS2 turned over both substrates well (Figure 6) [13].

The steady-state kinetic constants of the reactions were determined using spectrophotometric NADPH consumption assays (Table 1, Figure S1). The Km of SmIS1 for 8-oxogeranial was similar to the Km values previously reported for CrIS and OeIS, which are in the 1 μM range. However, the catalytic efficiency of SmIS1 was reduced compared with that of CrIS and OeIS. Compared with SmIS1, SmIS2 had lower affinity for 8-oxogeranial but higher affinity for NADPH.

### 2.3. Tissue Profile of SmIS1 and SmIS2 Accumulation

**Figure 7:**
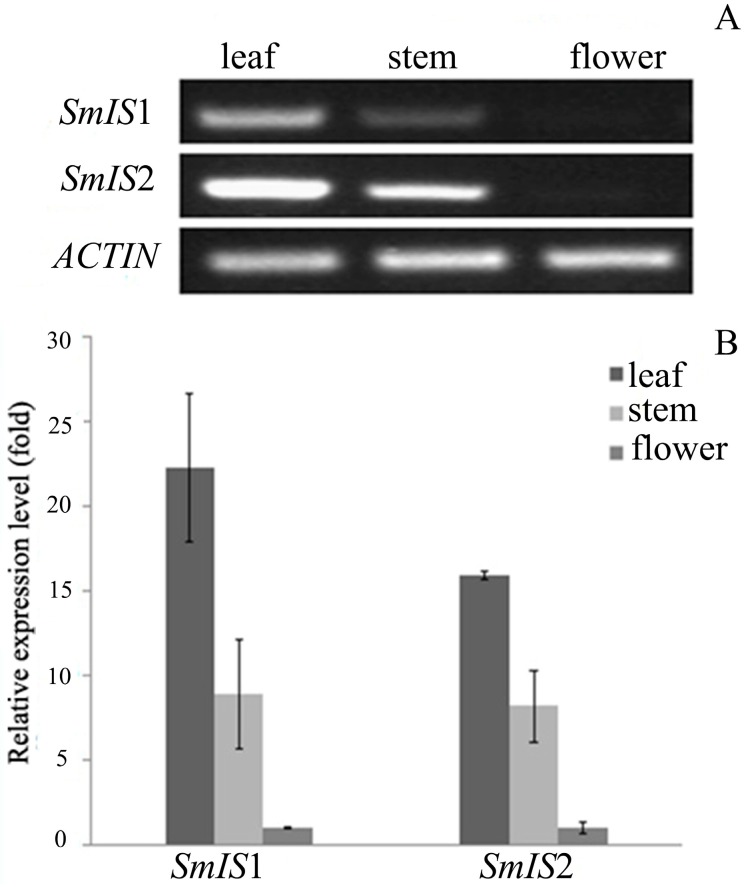
Expression pattern of the SmIS1 and SmIS2 genes in planta. (**A**) Semi-quantitative reverse transcriptase-polymerase chain reaction (RT-PCR) analysis in leaf, stems, and flower. Actin gene was used as a loading control; (**B**) Real-time quantitative PCR (RT-qPCR) analysis in leaf, stems, and flower.

The expression patterns of SmIS1 and SmIS2 were analysed in several *S. mussotii* tissues using semi-quantitative RT-PCR and RT-qPCR (Figure 7). The RNA transcripts of SmIS1 and SmIS2 were present in all of the organs that were tested and were more abundant in leaves and stems.

### 2.4. Prediction of Subcellular Localization of SmIS1 and SmIS2

We predicted the subcellular localization of SmIS1 and SmIS2 using WOLF PSORT, Predotar and ProtComp. We compared the prediction results in Table 2. WOLF PSORT predicted that SmIS1 was possibly localized to the endoplasmic reticulum or chloroplast. However, the corresponding scores were not high, and the Predotar and ProtComp predictions were inconsistent with the WOLF PSORT prediction results. The two programs predicted that SmIS1 was possibly localized to the cytoplasm. WOLF PSORT and ProtComp predicted that SmIS2 was possibly localized to the cytoplasm.

## 3. Discussion

*S. mussotii* plants grow mainly on the Qinghai Tibetan Plateau at altitudes greater than 3800 m, and their seeds germinate poorly when planted at low elevations [1]. Given that the entire plant is used in Tibetan medicine, overexploitation of this plant has resulted in limited wild resources [1]. Therefore, seeking other resources, e.g., growing the plant in other areas and/or developing organ or tissue culture systems, could represent a good strategy to address the gradual shortage in the *S. mussotii* plant supply. However, secondary metabolic pathways often change, and the reactive compounds in the cells or tissues of these systems accumulate much less compared to naturally growing plants. To regulate the production and accumulation of reactive compounds in the plant tissues, we should elucidate the related metabolic pathways, characterize genes involved in the metabolism and reveal their expression regulations.

Similar secondary metabolism pathways have been demonstrated in different plants, especially for the early biosynthetic steps. *C. roseus* has been used to characterize the secoiridoid biosynthesis pathway, and the gene encoding a key reaction step from 8-oxogeranial to iridoids, namely iridoid synthase (IS), has been recently identified [13]. IS genes belong to the P5βR gene family, and almost all P5βR proteins can reduce several substrates, such as progesterone, 8-oxogeranial and 2-cyclohexen-1-one, using NADPH as a co-substrate [15]. The homologous genes have also been studied in other species, underscoring their importance in secoiridoid biosynthesis [17,18].

In this study, based on the results of sequence and transcriptomic data mining in *S. mussotii*, we proposed the secoiridoid biosynthesis pathway (Figure 1). We also recognized three P5βR gene homologs and characterized two of these genes (SmIS1 and SmIS2). For the third homolog, we only obtained a partial mRNA sequence of 1008 nt, which exhibits 60.42% similarity to the corresponding portion of SmIS1 and SmIS2. As the 5’ end sequence is missing, we will clone it using 5′ RACE in the future.

Sequence and phylogenetic analyses demonstrated that the two SmIS genes belonged to the P5βRs group, the members of which convert progesterone to 5β-pregnane-3,20-dione in cardenolide-producing plants. This finding is in accordance with the statement that all IS proteins studied to date in cardenolide-free plants exhibit high sequence similarity with P5βRs [17].

The 3D model demonstrated strong conservation of the secondary structural elements between the modelled SmIS1 and SmIS2 proteins and CrIS and that the entire structure of IS is composed of a typical dinucleotide binding “Rossmann” fold (a characteristic feature of the SDR superfamily) [19,20] covered by a helical C-terminal extension (Figure 3). These results suggest that ISs are orthologues that are derived from a common ancestor in the P5βR family, which is similar to the findings reported in olive [17]. Further IS sequence phylogenetic analysis may elucidate how ISs have evolutionarily diverged from other P5βRs.

Both SmIS1 and SmIS2 catalyse the conversion of 8-oxogeranial, which demonstrates a redundancy of genes encoding IS enzyme activity in *S. mussotii*. IS gene redundancy in *C. roseus* has also been reported [15]. However, our enzyme assay analysis revealed that SmIS2 has approximately 8-fold reduced affinity than SmIS1 for 8-oxogeranial. In addition, phylogenetic analysis revealed that SmIS1 and SmIS2 were clustered in two groups. SmIS1 was clustered with ISs, whereas SmIS2 was clustered with P5βRs. These results suggested that SmIS2 could potentially be called SmP5βR1 and not be significantly involved in 8-oxogeranial conversion. Nevertheless, further experimental data are needed to elucidate its function in *S. mussotii* cell metabolism. The catalytic efficiency of SmIS1 was reduced compared with CrIS and OeIS. It is possible that the maltose-binding protein fusion observed in SmIS1 affected the conformation of SmIS1.

qRT-PCR results revealed that these two genes were co-expressed in three different *S. mussotii* tissues. The highest gene expression was noted in leaves. In a previous study, we also found that the transcript abundance of the GPPS gene was highest in leaves [21]. Although secoiridoids accumulate in all of the organs of the *S. mussotii* plant, the highest level was found in its flower [22]. This finding suggests the involvement of a transport mechanism wherein the metabolites are synthesized outside the storage tissue and later transported to floral tissue [23,24]. 

CrIS forms dimers or higher-order structures with exclusive cytosolic localization as assessed using fluorescent protein fusions and bimolecular fluorescence complementation assays [13]. In *S. mussotii*, based on prediction programs, both SmIS1 and SmIS2 are likely to be cytosolic (Table 2). Whether SmISs could also form homo or hetero dimers in *S. mussotii* requires future investigations.

## 4. Materials and Methods 

### 4.1. Plant Materials

*S. mussotii* seeds were obtained from Yushu County in Qinghai Province. *S. mussotii* plants were grown locally in a greenhouse at NanKai University in Tianjin, China. The greenhouse was maintained at 25 °C with a 16-h photoperiod. Various organs, including leaves, stems and flowers, were collected from one-year-old *S. mussotii* plants.

### 4.2. Cloning and Sequencing of S. mussotii SmIS1 and SmIS2 Genes

Total RNA was isolated from the leaves of *S. mussotii* plants using an Eastep Super Total RNA Extraction Kit (Promega, Shanghai, China) according to the manufacturer’s instructions and then eluted in RNase-free water. The quality, purity and concentration of the RNA was estimated spectrophotometrically using A260 and A280 measurements (NanoDrop, Thermo Scientific, Wilmington, MA, USA) followed by visualization on ethidium bromide-stained agarose gels. Quality was then assessed using agarose gel electrophoresis (Liuyi, Beijing, China). Transcriptome sequencing was performed by Novogene Bioinformatics Technology (Beijing, China) and the methods for data processing, assembly and annotation were described in [25]. The transcriptome analysis of *S. mussotii* will be published separately.

Based on the whole transcriptome data of leaves in *S. mussotii* before the flowering period, which were obtained using the BLAST similarity search program (http://www.ncbi.nlm.nih.gov/BLAST), the entire coding sequences of SmIS1 and SmIS2 were amplified using the primer pairs as shown in Table 3. cDNA synthesis was performed using PrimeScript^™^ RT Master Mix (Dalian TaKaRa, Dalian, China) and oligo dT primers. For PCR, we used cDNA as the template with TransStart FastPfu Fly DNA Polymerase (TransGen Biotech, Beijing, China). Thermal cycling was performed using the following program: one cycle of initial denaturation for 5 min at 94 °C, 30 cycles consisting 30 s at 94 °C, 30 s at 60 °C, and 90 s at 72 °C, and a final step for 10 min at 72 °C. The obtained PCR products were subcloned into the pEASY-Blunt Simple vector (TransGen Biotech, Beijing, China). The fragments were sequenced at the Beijing Genomics Institute (BGI), Shenzhen, China.

### 4.3. Sequence Alignment, Phylogenetic Analysis and Homology Modelling

A multiple sequence alignment of the amino acid sequences of the ISs was performed using DNAMAN software (version 7.0.2, Lynnon Corp., Pointe-Claire, QC, Canada) (www. lynnon.com). Phylogenetic analyses were performed using Molecular Evolutionary Genetics Analysis, version 7.0 (Biodesign Institute, Tempe, AZ, USA, http://www.megasoftware.net) [26] with the Neighbour-Joining method [27]. The robustness of the trees was tested by running 1000 bootstrap replications. Evolutionary distances were computed using the Jones-Taylor-Thornton model [28], and all positions that contained gaps and missing data were eliminated from the data set. Three-dimensional models of SmIS1 and SmIS2 were generated using the Swiss-model server (swissmodel.expasy.org) [29]. *C. roseus* CrIS (PDB: 5COB) was used as the template.

### 4.4. Heterologous Expression

The full-length ORF of *SmIS*1 cDNA was subcloned into the *BamH* I-*Hind* III sites of pETMALc-H (New England Biolabs, Ipswich, MA, USA). The resulting plasmid was used to express a fusion protein in which a maltose-binding protein was placed at the N-terminus. The full-length ORF of the SmIS2 cDNA was subcloned into the *Nde* I*-Xho* I sites of pET-28a (Novagen, Madison, WI, USA) to produce a fusion protein with 6-His tags at both the *N*-terminus and *C*-terminus. The constructed plasmids and the empty vector (used as a control) were transformed into *E. coli* BL21 (DE3) cells (Novagen, Madison, WI, USA). Correct in-frame insertion was verified using sequencing. A preculture (5 mL) was grown overnight at 37 °C in LB medium. This culture was used to inoculate 500 mL of fresh medium to a density with an OD600 of 0.4 at 25 °C. The cells were then grown for 12 h at 16 °C. Then, 0.3 mM isopropyl-β-D-thiogalactopyranoside (IPTG) was added to induce protein expression. The cells were harvested using centrifugation and disrupted using sonication. The soluble maltose-binding protein-tagged SmIS1 protein was then purified to apparent homogeneity as previously described by Gavidia et al. [30].

The SmIS2 protein was purified from the resulting soluble fraction using the His Trap FF (GE Healthcare, Hammersmith, UK). The concentration of the purified protein was quantified using the Bradford method, and the proteins were visualized using SDS–PAGE (Liuyi, Beijing, China).

### 4.5. GC-MS-Based Assays

The enzyme assays were performed essentially as previously described [13] using 20 mM MOPS (pH = 7.0) as a buffer. The protein concentration was set to 0.05 mg/mL. The substrate concentration was 0.03 mM. The NADPH concentration was 0.4 mM, and the final assay volume was 1 mL. The reaction was terminated after 1 h by adding 1 mL of dichloromethane at 25 °C. The reaction products were extracted, and the organic phase was evaporated at 25 °C. For the GC-MS analysis (Agilent, Palo Alto, CA, USA), the samples were dissolved in 100 μL of dichloromethane. 8-Oxogeranial was purchased from Toronto Research Chemicals (TRC) (Toronto Research Chemicals, Toronto, ON, Canada).

GC-MS analysis was performed in an Agilent 7890 A System coupled to an Agilent 5975 C MS detector (Agilent, Palo Alto, CA, USA). A J & W GC column (30 m × 0.25 mm × 0.25 μm) and helium gas (1.2 mL/min) were used. The program started at 60 °C, and the temperature was increased 5 °C/min to 150 °C, 20 °C/min to 240 °C, 20 °C/min to 290 °C and then maintained for 5 min at 290 °C.

### 4.6. Enzyme Kinetics of SmIS1 and SmIS2

SmIS1 and SmIS2 enzymatic activity was examined in 20 mM MOPS buffer (PH 7.0). Monoterpene substrates were stored as 50 mM stocks in tetrahydrofuran (THF) at −20 °C. Reductase activity was measured using a microplate reader (PerkinElmer, Waltham, USA). The conversion of NADPH to NADP^+^ was monitored at 340 nm over a time course of 5 min at 40 °C. The following reaction system was used: varying amounts of NADPH (0–100 mM) and substrate (0–70 mM). SmIS1 and SmIS2 proteins concentrations were set as 0.014 mg/mL and 0.02 mg/mL, respectively, in a final assay volume of 250 μL. Km and Vmax were calculated using the Michaelis-Menten equation with a non-linear regression. The Kcat value was calculated by dividing V_max._ by Et (the number of enzymes in each assay).

### 4.7. Transcript Analysis

RNA was isolated from different *S. mussotii* tissues using an Eastep Super Total RNA Extraction Kit (Promega, shanghai, China). After reverse transcription was performed, the cDNAs for *Actin* (used as a control), SmIS1 and SmIS2 were amplified using RT-PCR with specific primers (Table 3) [Note: The same specific primers were used in both the RT-PCR and real-time quantitative PCR (RT-qPCR) analyses]. The following program was used: 94 °C for 5 min, 30 cycles at 94 °C for 30 s, 60 °C for 30 s and 72 °C for 1 min, and a final incubation cycle at 72 °C for 10 min. The RT-PCR products were electrophoresed on 1 % agarose gels and visualized under UV light using ethidium bromide. RT-qPCR was performed on an iCycler Thermal Cycler (Bio-Rad iQ5, Hercules, CA, USA) using SYBR Premix Ex Taq II (Dalian Takara, Dalian, China) according to the standard manufacturer’s protocol. The reaction mixture was heated to 95 °C for 30 s followed by 40 PCR cycles at 95 °C for 5 s, 58 °C for 30 s and 72 °C for 30 s. All primer pair efficiencies were between 95% and 105%, and the individual efficiency values were considered in the calculation of normalized relative expression. The difference in the relative expression levels of *SmIS*1 (or *SmIS*2) were calculated using the 2^−Δ^^Δ^^CT^ method after the data were normalized to *actin*. All values are shown as the mean ± standard error of the mean using at least three biological replicates.

### 4.8. Subcellular Localization Prediction

We predicted the subcellular localization of SmIS1 and SmIS2 using WOLF-PSORT tool (http://www.genscript.com/wolf-psort.html), Predotar (http://urgi.versailles.inra.fr), and ProtComp (http://www.softberry.com/) [31,32,33].

## 5. Conclusions

In conclusion, iridoid synthases (SmIS1 and SmIS2) were isolated from *S. mussotii* and then heterologously expressed and biochemically characterized for the first time. These two proteins contained the same specific motifs and conserved active amino acids that have been reported in other P5βRs obtained from cardenolide plants or cardenolide-free plants. The two purified *E. coli*-expressed proteins reduced 8-oxogeranial to both nepetalactol and iridodials. This result confirmed the notion that ‘IS activity’ is indeed an intrinsic capacity of angiosperm P5βR proteins. In the future, we will analyse the functions of the two genes in the secoiridoid synthetic pathway of *S. mussotii* using gene silencing. In addition, we will identify more candidate genes in the secoiridoid synthesis pathway using transcriptome data analysis. The current investigation has added to our knowledge base of P5βRs genes in the secoiridoid synthesis pathway and provided candidate genes for genetically improving *S. mussotii* by enhancing its secondary metabolite production.

## Figures and Tables

**Table 1:** Compared kinetic parameters of recombinant of SmIS1 and SmIS2.

Enzyme	Substrate					
	**8-Oxogeranial**			**NADPH**		
	**Km**	**Kcat**	**Kcat/Km**	**Km**	**Kcat**	**Kcat/Km**
	**(μM)**	**(s^−1^)**	**(s^−1^M^−1^)**	**(μM)**	**(s^−1^)**	**(s^−1^M^−1^)**
SmIS1	7.29 ± 1.59	0.11 ± 0.007	15,559.6 ± 4354.3	77.94 ± 6.03	0.135 ± 0.01	1731.7 ± 11.3
SmIS2	54.37 ± 4.29	0.034 ± 0.001	628.6 ± 75.9	41.83 ± 7.2	0.026 ± 0.01	628 ± 104.7

The data represent the mean of three independent measurements ± SD. (*n* = 3).

**Table 2:** Prediction of SmIS1 and SmIS2 subcellular localization using website programs.

Enzyme	Subcellular Localization Prediction Methods					
	WOLF PSORT		Predotar		ProtComp	
SmIS1	ER	6	Mito	0.09	Cyto	8.44
	Chlo	5	Plastid	0.01	ER	0.13
	Mito	1	ER	0	Chlo	0.84
	Plastid	1	Else	0.9	Vacuolar	0.38
SmIS2	Cyto	9	Mito	0.01	Cyto	8.32
	Cysk	3	Plastid	0.01	ER	0.27
	Plastid	1	ER	0	Chlo	0.82
			Else	0.98	Vacuolar	0.43

For WoLF PSORT, Predotar and ProtComp predictions, the favourable localizations are reported with corresponding scores. ER, endoplasmic reticulum; Chlo, chloroplast; Mito, mitochondria; Cyto, cytoplasm; Cysk, cytoskeleton.

**Table 3:** Primers used in this study.

Primer usage	Gene	Forward (5′-3′)	Reverse (5′-3′)
ORF Cloning	SmIS1	gcggatccATGAGCTGGTGG	cccaagcttCTATGGGATAAATTTG
		TGGAAAAG	AAGTCTCTC
	SmIS2	ggaattccatATGAGCTGGTG	ccctcgagAGGAACAATTTTATGA
		GTGGACTGGT	GCTTTCATT
RT-PCR	SmIS1	AAGCATGAAAAAAAGC	CAATATATCCCACCATTTCCAT
		CATTAGT	
	SmIS2	AAGCATGAAAATGTTCC	ATTCTCGTCGAATTCCACGTA
		TTTCAC	
	Actin	ACTGGTGTTATGGTTGG	TCGGTGAGAAGTATAGGGTGCGG
		TATTGG	
qRT-PCR	SmIS1	AAGCATGAAAAAAAGC	CAATATATCCCACCATTTCCAT
		CATTAGT	
	SmIS2	AAGCATGAAAATGTTCC	ATTCTCGTCGAATTCCACGTA
		TTTCAC	
	Actin	ACTGGTGTTATGGTTGG	TCGGTGAGAAGTATAGGGTGC
		TATGG	

Lower case letters indicate the protective bases and restriction enzymes sites.

## Supplementary Materials

Supplementary materials are available online.
