# Supplementary material for: Cloning and Characterization of Two Iridoid Synthase Homologs from Swertia Mussotii
Source: Molecules. 2017 Aug 22;22(8):1387. doi: 10.3390/molecules22081387 (PMC6152284; doi:10.3390/molecules22081387)
Supplement: Supplementary file 1 [file molecules-22-01387-s001.pdf]

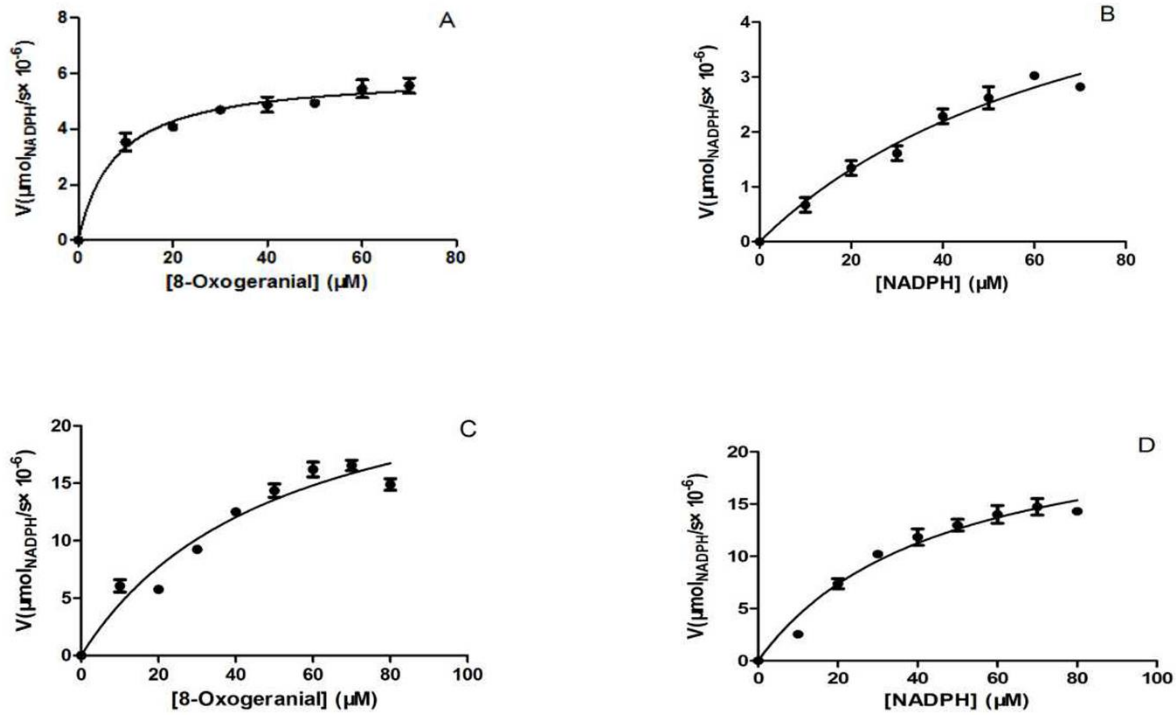

Figure S1. Steady-state kinetic analysis of the iridoid synthase reaction. (A) Saturation curve of SmIS1 for 8-oxogeranial at a fixed NADPH concentration of 100 μM. (B) Saturation curve of SmIS1 for NADPH at a fixed 8-oxogeranial concentration of 30 μM. (C) Saturation curve of SmIS2 for 8-oxogeranial at a fixed NADPH concentration of 100 μM. (D) Saturation curve of SmIS2 for NADPH at a fixed 8-oxogeranial concentration of 30 μM. Reaction rates were measured spectrophotometrically, monitoring NADPH consumption at 340 nm. Individual data points are averages of three replicates. Error bars represent standard errors (SE).
